# A Summary Measure of Health Inequalities for a Pay-for-Population Health Performance System

**Published:** 2010-06-15

**Authors:** Yukiko Asada

**Affiliations:** Department of Community Health and Epidemiology, Faculty of Medicine, Dalhousie University

## Abstract

A system that rewards population health must be able to measure and track health inequalities. Health inequalities have most commonly been measured in a bivariate fashion, as a joint distribution of health and another attribute such as income, education, or race/ethnicity. I argue this practice gives insufficient information to reduce health inequalities and propose a summary measure of health inequalities, which gives information both on overall health inequality and bivariate health inequalities. I introduce 2 approaches to develop a summary measure of health inequalities. The bottom-up approach defines attributes of interest, measures bivariate health inequalities related to these attributes separately, and then combines these bivariate health inequalities into a summary index. The top-down approach measures overall health inequality and then breaks it down into health inequalities related to different attributes. After describing the 2 approaches in terms of building-block measurement properties, aggregation, value, data and sample size requirements, and communication, I recommend that, when data are available, a summary measure should use the top-down approach. In addition, a strong communication strategy is necessary to allow users of the summary measure to understand how it was calculated and what it means.

## Introduction

Developers of any performance reward system must select the performance improvements that deserve rewards and ensure fairness by measuring them appropriately. Measurement is arguably more challenging in pay-for-performance systems that reward population health than those that reward medical care because determinants of population health go beyond medical care. The questions sketched by Kindig (1) summarize challenges of measurement in a pay-for-performance system that rewards population health: 1) How should we measure health outcomes?, 2) How should we measure health inequalities?, and 3) How should we balance the need for improvement in both?

This article focuses on the second question and calls for development of a summary measure of health inequalities, where health inequalities associated with multiple attributes (such as income, education, and race/ethnicity) are summarized into 1 number. I assume typical measures of population health, such as life years or health-adjusted life years, and population units that have a mandate for the health of their population, such as states. However, the core idea of a summary measure presented here can in principle be applied to other measures of population health and other population units.

## Background

Because health inequality is an established field of research and policy making, we might expect that a well-tested template would be available for measuring health inequalities that could be used in a pay-for-population health performance system. However, such guidance has not yet been established. Over the past century, many empirical studies have described health inequalities (2,3), and useful guides for measuring health inequalities are now available (4,5). In the past few decades, jurisdictions and organizations have endorsed reducing health inequalities (6) and have focused their efforts accordingly. The World Health Organization's (WHO's) Commission on Social Determinants of Health (7) is a notable example of such concerted efforts. Despite these efforts, progress has been inadequate in reducing health inequalities. One reason could be the lack of an effective strategy to measure and track health inequalities.

**Figure 1:**
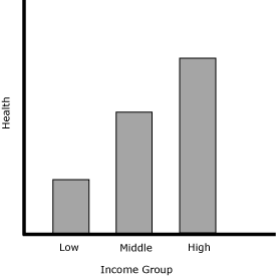
A hypothetical presentation of a bivariate health inequality. Measures of bivariate health inequality assess the association of health inequality with another attribute, in this example, income.

Health inequalities have most commonly been measured in a bivariate fashion, as a joint distribution of health and another attribute, such as income, education, sex, or race/ethnicity (8). A typical measure of bivariate health inequality assesses 1 attribute at a time, for example, different levels of health across income groups (Figure 1). The degree of health inequality across groups can be quantified by an index such as a range measure that compares the health of 2 groups (5). A more sophisticated approach assesses the level of income (or another attribute) for each individual rather than the average level of health of each group. An index that quantifies the degree of inequality can be complex, for example, the Concentration Index, which compares the health of every individual or income group (5). Regardless of the unit of analysis (group or individual) or the inequality index used, measures of bivariate health inequalities always assess health inequality in relation to another attribute.

**Figure 2:**
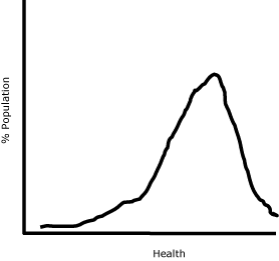
A hypothetical presentation of a univariate health inequality. Measures of univariate health inequality assess health inequality across individuals regardless of its association with other attributes.

Around 2000, there was a brief but heated debate about whether we should continue to measure bivariate health inequalities or start measuring univariate health inequality (9-13). Regardless of their association with other attributes, measures of univariate health inequality assess health inequality across individuals in the same way that income inequality is typically assessed (Figure 2). A few researchers had measured health inequalities in a univariate fashion (14-16), but Murray and colleagues proposed univariate health inequality as the best focus in the assessment of population health (10,17,18).

This debate raised moral and policy questions (19). Health has an intrinsic importance, those who support measuring univariate health inequality argued, and we should not only be interested in health inequality by socioeconomic status, as most studies have focused on, but also in how health itself is distributed. The supporters of measuring bivariate health inequalities believed that health inequalities are significant when they are associated with other attributes, such as income. Simply put, with an example of income, this debate was about whether we should be worried about sick people regardless of their income level (univariate health inequality), or about impoverished sick people more than the wealthy sick people (bivariate health inequality).

Furthermore, those who support measuring univariate health inequality argued that the choice of which attributes to study is generally driven by the investigator's intuition or interest. Accordingly, we now have numerous empirical descriptions of health inequalities by various attributes, which are not necessarily comparable and do not immediately offer an overall picture of health inequalities. Univariate health inequality, they maintained, can offer an overall picture of health inequality in the population in a way that is comparable across populations. The advocates of measuring bivariate health inequalities, on the other hand, argued that univariate health inequality does not suggest how to tailor interventions or policies to reduce health inequalities.

The result of this debate was an acknowledgment — primarily from supporters of univariate health inequality — that bivariate and univariate health inequalities are complementary (though exactly how they are complementary has not been specified) (20-22). Most empirical work has continued to measure bivariate health inequalities. Regarding univariate health inequality as a rarely used alternative, however, is a missed opportunity for health inequality research and policy. This debate points to a need for a better strategy to measure and track health inequalities.

This debate also suggests a strong resistance among health inequality researchers to abandoning bivariate health inequalities. They may be resistant because 1) they view health as not only intrinsically important but also as valuable in terms of its associations with other attributes, and 2) it is useful to know who is sick in order to develop policies. Arguments for measuring univariate health inequality also have merit. Lack of comparability of results and an overall view of health inequalities may be a barrier between numerous descriptions of health inequalities and effective policy making. A lesson from this debate may be that we need to develop a summary measure of health inequalities, which gives an overall picture of health inequalities in the population while maintaining pertinent information on bivariate health inequalities.

## Two Approaches for a Summary Measure of Health Inequalities

Relevant literature suggests 2 approaches to developing a summary measure of health inequalities: the bottom-up and top-down approaches.

### The bottom-up approach

The bottom-up approach first defines attributes of interest and measures bivariate health inequalities related to these attributes separately. It then combines these bivariate health inequalities into a summary index. An example is the inequality measure developed for the *Health of Wisconsin Report Card 2007* (hereafter, the "Wisconsin inequality measure") (23,24). The Wisconsin inequality measure extends the Index of Disparity (25,26), a modified coefficient of variation defined as equation no. 1.

Equation 1

ID=∑J=1J−1∣rj−rref∣Jrref(x)100

Where* r_j_* is health of the *j*th group, *r_ref_* is health of the reference group, and *J* is the number of groups compared. The Index of Disparity is the average deviation of the health of groups compared with the reference group's health, expressed as a percentage. When all groups have the same health, the index value is 0. Higher values suggest more inequality.

**Figure 3:**
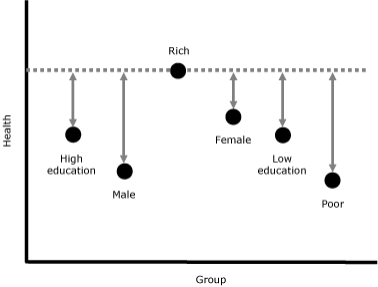
A simplified example of the Wisconsin health inequality measure. To obtain the overall health inequality, calculate the difference from the reference health level (rich) for each group (poor, low education, high education, male, and female), sum them, and divide by the number of groups minus 1 (6 − 1 = 5).

The Wisconsin inequality measure calculated the Index of Disparity by using all 14 groups (2 sex groups, 3 education groups, 4 rurality groups, and 5 race/ethnicity groups) and converted the index to a letter grade for ease of communication. All attributes (sex, education, rurality, and race/ethnicity) are considered to be of equal importance. The reference is set as the best health level among all groups (Figure 3).

### The top-down approach

The top-down approach first measures univariate health inequality, then breaks it down into health inequalities related to different attributes. Unlike the bottom-up approach, there is no known example of a summary measure of health inequalities using this approach. However, this approach comes close to the principal idea underlying WHO's health inequality measurement in the *World Health Report 2000* (17,18), and similar methods have been proposed in other contexts. For example, this approach is similar to the framework of unfair inequalities in health and health care proposed by Fleurbaey and Schokkaert (27), although they do not propose it for a summary measure. It is also akin to inequality measure decomposition by attributes, though in health research this technique is most often used with the Concentration Index (28), a sophisticated measure of bivariate health inequality. Using decomposition, we can tell which attributes (eg, education and sex) explain a bivariate health inequality (eg, income-related health inequality) and to what degree. Although the Concentration Index decomposition is a useful tool to understand bivariate health inequality, it is different from decomposing univariate health inequality as a summary measure.

The top-down approach first attempts to explain the level of health of individual *i* by determinants of health. In the simplest form, Fleurbaey and Schokkaert define such a "structural model" as equation no. 2.

Equation 2

*h_i _* = F(*N_i_*, *S_i_*, *I_i_*, *P_i_*, *Z_i_*)

Where* N* is biologically determined health endowments, *S* is social background, *I* is available information, *P* is individual preferences, and *Z* is health care supply. At the risk of a gross simplification, empirically, *N* might be captured by age, *S* by income, *I* by education, *P* by health behavior such as smoking, and *Z* by health insurance. Variables can be extended to the community level, for example, adding neighborhood income for *S*, and rurality for *Z*. The top-down approach then asks which of these determinants or attributes are, following the increasingly used term in health economics, "illegitimate" or result in unfair inequality across individuals. For some attributes, there is a consensus on this question. For example, health inequality associated with social background typically is considered unfair. The top-down approach measures the distribution of *h_i_* (univariate health inequality) and identifies the contribution of each of the illegitimate attributes, however, defined, to univariate health inequality. Figure 4 is an example of information that the top-down approach can give.

**Figure 4:** An example of information given by the top-down approach. The top-down approach provides information on univariate health inequality (as overall health inequality) and identifies contributions of the attributes we select (eg, income, education, and race/ethnicity). "Other (residual)" shows univariate health inequality that is not associated with the chosen attributes.

**Table:** 

**Attribute**	**Degree of Health Inequality**	**% Contribution**
Overall		
Income		
Education		
Race/ethnicity		
Other (residual)		

## Issues for Developing a Summary Measure of Health Inequalities

Which approach is better suited to develop a summary measure of health inequalities? To answer this question, I address the following 5 issues: building blocks, aggregation, value, data and sample size requirements, and communication. Building blocks are common to both the bottom-up and top-down approaches. The subsequent 4 issues separate these 2 approaches.

### Building blocks

Whichever approach we take, we should carefully choose a bivariate or univariate measure that becomes a building block of a summary measure. The building block for the Wisconsin inequality measure, an example of the bottom-up approach, is the Index of Disparity, and the Gini coefficient (5) can be used as a building block for the top-down approach. To decide whether they are appropriate building blocks on which to base a summary measure, we must examine the questions researchers ask when choosing health inequality measures (Table 1) (4,5).

**Figure 5:**
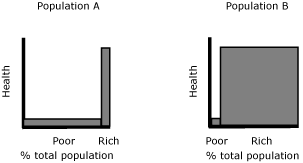
Inequality judgment and subgroup population size. The width of the bars suggests the proportion of poor and rich people in the 2 populations. If we consider the degree of income-related health inequality differs in these populations, an inequality measure should be sensitive to this difference.

All measurement properties of the Index of Disparity and the Gini coefficient coincide with the current discussion (4,5), except sensitivity to the mean (both measures) and subgroup considerations (Index of Disparity) (Table 1). The literature often recommends that researchers use both an absolute (ie, translation invariant) and a relative (ie, scale invariant) measure (5). This recommendation reflects the lack of consensus among researchers on the issue of sensitivity to the mean. However, researchers should choose one after trying both measures and understanding the nature and limitation of the chosen measure. Policy makers and the general public should not be given 2 measures (and possibly two different answers) without guidance. Insensitivity to the group size of the Index of Disparity contradicts the recommendation in the health inequality literature (4,5). Measuring bivariate health inequality with the Index of Disparity, we would consider the 2 populations in Figure 5, with 2 groups of different sizes, have the same degree of inequality. We may judge that the degrees of health inequality in these 2 populations are different because, for example, suffering is likely to be more prevalent in Population A than in Population B, given its larger proportion of poor people (4). In this case, bivariate inequality measures should be sensitive to group size because a measure of inequality should reflect our perception of inequality. Sensitivity to the group size, in practice, can be incorporated in the measure by giving a proportional weight to each group (5).

### Aggregation

The bottom-up and top-down approaches aggregate bivariate inequalities to overall health inequality differently. The bottom-up approach aggregates bivariate inequalities arbitrarily, and the top-down approach decomposes univariate inequality into bivariate inequalities. This difference has 3 implications. First, the top-down approach can identify an independent association between each attribute and health and also interactive associations between attributes and health. Although possible, identifying independent and interactive effects is cumbersome in the bottom-up approach. The bottom-up approach starts by measuring unadjusted bivariate health inequalities, where each attribute of health inequality is measured without consideration for other attributes. We can categorize groups further, for example, from rich and poor (income) and male and female (sex) to rich male, rich female, poor male, and poor female. However, this is a time-consuming way to describe independent and interactive effects of multiple determinants of health.

Second, the difference in aggregation between the 2 approaches leads to a difference in the meaning of an overall picture of health inequalities. An overall health inequality is a composite in the bottom-up approach, but it is univariate health inequality in the top-down approach. The top-down approach has a logical and mathematical hierarchy from bivariate health inequalities to univariate health inequality; the sum of bivariate health inequalities equals univariate health inequality. The bottom-up approach does not have such a hierarchy. Because each individual in the population belongs to multiple groups (eg, an individual is female, rich, educated, and minority), it is unclear exactly what an aggregation of non-mutually exclusive bivariate health inequalities means.

Finally, by decomposing univariate health inequality into bivariate health inequalities, the top-down approach can identify the contribution of each bivariate health inequality to univariate health inequality and thus the relative importance of bivariate health inequalities. For example, Wagstaff and van Doorslaer (29) reported that income-related health inequality accounted for approximately 25% of univariate inequality in malnutrition among Vietnamese children and general health status among Canadian adults, by using a subgroup decomposition technique that focuses on 1 attribute (as opposed to multiple attributes, as I am proposing here). Because of the use of a composite to indicate overall health inequality, the bottom-up approach cannot identify the relative contribution of each bivariate attribute.

### Value

A measure can be descriptive (describing the object) or normative (incorporating our value of the object). Using either the bottom-up or top-down approach, a summary measure of health inequalities is normative in the most fundamental sense; it measures health inequalities that we value. But these approaches differ in terms of how normativity is introduced, and the top-down approach offers a richer framework than the bottom-up approach. The bottom-up approach starts by selecting attributes that we believe to be important in relation to health inequality. The top-down approach, on the other hand, starts by describing health inequalities and moves on to normative assessment of fair and unfair health inequalities (27). This assessment is done by selecting attributes that we believe to cause unfair health inequalities, and the top-down approach can embed the reasons these attributes are important, as Fleurbaey and Schokkaert suggest in the formation of *N* (health endowments), *S* (social background), *I* (available information), *P* (individual preferences), and *Z* (health care supply) (27). These selections and considerations can be incorporated in the bottom-up approach but are not built into it.

Furthermore, in either approach we must ask whether a summary measure of health inequalities should incorporate the relative importance of different attributes. According to Wagstaff and van Doorslaer (29), income-related health inequality explains approximately 25% of overall, univariate health inequality. If we believe that income-related health inequality is more important than other bivariate health inequalities (eg, education-, sex-, or geography-related health inequalities), then we might wish to reflect our value in the measurement by giving more weight to income-related health inequality than 25%. The Wisconsin inequality measure treats all bivariate health inequalities as equally important. The top-down approach describes the contribution of each attribute to univariate health inequality without considering which attribute is more important than others. If we wish to develop a summary measure of health inequalities to incorporate the importance of different attributes, whose values should be included and in what way? What about concentration of burden? We may not merely consider 1 attribute to be more important than another but multi-attribute correlations (for example, the sick who are poor, uneducated, and a minority) to be morally problematic. Not surprisingly, given the uncoordinated numerous descriptions of bivariate health inequalities, the current empirical health literature is silent about these value questions.

### Data and sample size requirements

Generally, the top-down approach requires more data than the bottom-up approach. The top-down approach works best with individual-level data on health and determinants of health, while the bottom-up approach can be pursued with group-level data. Population health surveys, possibly linked with census data, may offer enough information for the top-down approach, but the sample size of the survey determines how small the population can be for which a summary measure of health inequalities can be calculated. Despite the clear advantage of the top-down approach in terms of aggregation and value, data and sample size requirements may be a critical hindrance to its policy application.

These considerations for data and sample size requirements are typical in any quantitative analysis, but the use of a summary measure of health inequalities for a system of pay-for-population health performance requires at least 2 further considerations. First, how sensitive should a summary measure be to changes? If we agree to reward performance in the short term (eg, in 3-5 years), a summary measure should be sensitive to changes that occur in this time frame, and data should be updated regularly. Second, for which population (eg, state, county, community) does it make the most sense to establish a pay-for-performance system? The smallest population for which data are available may not necessarily be the most appropriate size.

### Communication

Effective use of a summary measure of health inequalities demands clear communication. Ideally, a measure should be conceptually and methodologically sound and easy to communicate. The bottom-up approach is arguably methodologically simpler than the top-down approach. However, ease of communication does not necessarily equal simplicity in concepts and methods. A complex Concentration Index decomposition, similar to the top-down approach, has been increasingly used in policy-oriented work (28). Complex concepts and methods require an effective communication strategy.

I suggest a summary measure of health inequalities using the top-down approach and a strong communication strategy when data and sample size requirements are surmountable. Compared with the bottom-up approach, it offers a conceptually clearer meaning of overall health inequality and a richer framework for choosing relevant attributes associated with health inequality. In addition, development of a summary measure of health inequalities requires clarification of value questions.

## Recommendations

First, a system of pay-for-population health performance should incorporate measurement of health inequalities. Second, measurement of bivariate health inequalities, the most common way to measure health inequalities, may not be the most effective mechanism to reduce health inequalities. A system that rewards population health should seek to develop a summary measure of health inequalities. Third, a summary measure of health inequalities can be developed by adopting the bottom-up or top-down approach. When data are available, a summary measure using the top-down approach should be used, along with a strong communication strategy to help users understand what the measure means and how it was calculated. Finally, clarification of value questions is a high priority for development of a summary measure of health inequalities.

## Figures and Tables

**Table.** Questions That Arise in Selecting Health Inequality Measures and Measurement Properties of the Index of Disparity and the Gini Coefficient

Question	Index of Disparity	Gini Coefficient
**Comparison**		
Who is compared against whom or what? Should the comparison be made in terms of health only (univariate) or health and another attribute (bivariate)?	The healthiest group against all other groups	Everyone against everyone
**Aggregation**		
How are differences aggregated at the population level? For bivariate health inequality measures, should the measures be sensitive to inherent ordering of another attribute (eg, income)?	Unweighted addition of difference and sensitive to inherent ordering of attribute	Weighted addition of health share and unweighted addition of difference
**Sensitivity to the mean**		
Should the judgment of inequality be sensitive to the mean level of the population? Absolute measures are translation invariant, meaning that equal absolute difference implies equal degree of inequality, while the equal proportional increase makes inequality larger. Relative measures are scale invariant, meaning that equal proportional difference implies equal degree of inequality, while the equal absolute addition reduces inequality. Intermediate inequality measures consider equal proportional increase makes inequality bigger, while equal absolute addition decreases inequality.	Translation invariant	Scale invariant
**Sensitivity to the total population size**		
Should the judgment of inequality be sensitive to the total population size?	Insensitive	Insensitive
**Subgroup considerations**		
Should the judgment of inequality be sensitive to the subpopulation size? How should the overall inequality of a population correspond to inequalities of subgroups in that population?	Insensitive to the group size	Decomposable
